# Preoperative Seizure Control and Perioperative Anesthetic Outcomes in Children Receiving Levetiracetam Versus Phenytoin: A Hospital-Based, Prospective, Comparative Cohort Study

**DOI:** 10.7759/cureus.106556

**Published:** 2026-04-06

**Authors:** Anshika Agarwal, Priyanka V Shah, Parth S Shah, Sunil Pathak, Ramlakhan Meena

**Affiliations:** 1 Obstetrics and Gynaecology, Private Practice, Datia, IND; 2 Pediatrics, U.N. Mehta Civil Hospital, Ahmedabad, IND; 3 Pediatrics, BJ Medical College and Civil Hospital, Ahmedabad, IND; 4 Pediatrics, Dr. N.D. Desai Faculty of Medical Science and Research, Dharmsinh Desai University, Nadiad, IND; 5 Community Medicine, Government Medical College, Datia, IND

**Keywords:** anesthetic recovery, case–control study, epilepsy, levetiracetam, pediatric anesthesia, perioperative seizures, phenytoin

## Abstract

Background and objective

Epilepsy is a common neurological disorder in children, and the perioperative period is associated with an increased risk of seizures and anesthetic complications. Intravenous phenytoin loading is associated with hemodynamic instability, whereas chronic oral therapy has minimal intraoperative cardiovascular effects. In contrast, levetiracetam has more favorable pharmacokinetics and fewer drug interactions. However, evidence comparing perioperative outcomes between levetiracetam and phenytoin in elective pediatric surgical settings remains limited. This study aimed to compare perioperative seizure incidence and anesthetic outcomes between children receiving levetiracetam and phenytoin and to identify predictors of postoperative seizures.

Methods

This hospital-based, prospective, comparative cohort study was conducted over 18 months at a tertiary care center. Two hundred children aged 1-18 years with epilepsy undergoing elective non-neurosurgical procedures under general anesthesia were enrolled, with 100 receiving levetiracetam and 100 receiving phenytoin monotherapy. Antiepileptic therapy was continued perioperatively. The primary outcome was the occurrence of postoperative seizures within 24 hours. Secondary outcomes included preoperative seizure-free status, intraoperative and postoperative complications, delayed emergence, recovery time, and length of hospital stay. Data were analyzed using appropriate statistical tests and multivariable logistic regression analysis to adjust for confounders.

Results

Baseline characteristics were comparable, except for a higher proportion of patients who were seizure-free preoperatively in the levetiracetam group (79% vs. 54%, p < 0.001). Postoperative seizures occurred in 6% of the levetiracetam group and 12% of the phenytoin group (p = 0.138). Delayed emergence was significantly lower with levetiracetam (7% vs. 19%, p = 0.012). Recovery time (34.5 ± 9.1 vs. 51.4 ± 12.8 minutes) and hospital stay (2.95 ± 0.86 vs. 4.83 ± 1.39 days) were significantly shorter in the levetiracetam group. After adjustment, preoperative seizure-free status showed a protective trend, whereas the type of antiepileptic drug (AED) was not independently associated with postoperative seizures.

Conclusions

Levetiracetam and phenytoin demonstrated comparable postoperative seizure incidence after adjustment for confounding factors. However, levetiracetam was associated with improved perioperative recovery, including reduced delayed emergence and a shorter hospital stay. These findings suggest that while the efficacy of seizure prevention may be similar, differences in pharmacological profiles may influence perioperative safety and recovery outcomes in pediatric surgical patients.

## Introduction

Epilepsy is one of the most common chronic neurological disorders worldwide, affecting approximately 50 million individuals and representing a major source of neurological morbidity in children [1]. Children with epilepsy frequently undergo elective surgical procedures under general anesthesia. The perioperative period may increase seizure susceptibility due to physiological stress, fasting, sleep deprivation, altered antiepileptic drug (AED) levels, and potential interactions between anesthetic agents and AEDs [2]. Inadequate perioperative seizure control has been associated with intraoperative seizures, delayed emergence from anesthesia, prolonged hospitalization, and increased intensive care utilization, highlighting the importance of optimal preoperative AED management [3].

Phenytoin, a conventional AED, remains widely used but is characterized by nonlinear pharmacokinetics, hepatic enzyme induction, and significant drug-drug interactions that may complicate anesthetic care [4]. Intravenous phenytoin loading has been associated with hemodynamic instability, including hypotension and arrhythmias, whereas chronic oral therapy typically has minimal intraoperative cardiovascular effects. In contrast, levetiracetam exhibits minimal protein binding, negligible hepatic metabolism, limited drug interactions, and favorable tolerability, making it an attractive alternative in perioperative settings [5]. Differences in pharmacological profiles may also influence perioperative airway and respiratory events through mechanisms such as altered anesthetic metabolism, enhanced sedation, and respiratory depressant effects.

Several pediatric studies have demonstrated comparable or improved seizure control with levetiracetam compared with phenytoin, particularly in emergency and neurocritical contexts such as convulsive status epilepticus and neurosurgical seizure prophylaxis [6,7]. However, these investigations have primarily focused on seizure cessation or early postoperative seizure prevention in high-risk neurosurgical populations. They rarely evaluate children undergoing elective non-neurosurgical procedures and provide limited assessment of intraoperative hemodynamic stability, airway complications, delayed emergence, respiratory depression, and overall recovery parameters. In addition, few studies have adjusted for baseline seizure control status or explored independent predictors of postoperative seizures using multivariable analysis.

These limitations restrict the applicability of existing evidence to routine elective pediatric surgical practice. Determining whether the choice between levetiracetam and phenytoin influences not only seizure prevention but also anesthetic stability and recovery outcomes is clinically important. Identification of an agent associated with improved perioperative safety and faster recovery could reduce complications, shorten hospitalization, and optimize healthcare resource utilization. Therefore, the present prospective, comparative cohort study aimed to compare perioperative seizure incidence between children receiving levetiracetam and phenytoin, evaluate differences in preoperative seizure control and perioperative anesthetic outcomes, and identify independent predictors of postoperative seizures using multivariable analysis.

## Materials and methods

Study design and setting

This hospital-based, prospective, comparative cohort study was conducted in the Departments of Pediatrics and Anesthesiology at a tertiary-care teaching hospital in Central India over a period of 18 months. The study was designed to evaluate the association between preoperative AED therapy and perioperative anesthetic outcomes in children undergoing elective surgery under general anesthesia.

Study population

Children aged 1 to 18 years with a confirmed diagnosis of epilepsy who were scheduled for elective surgery under general anesthesia were consecutively screened in the preoperative clinic. Participants were eligible if they had a history of epilepsy for at least six months and were receiving stable monotherapy with either levetiracetam or phenytoin for a minimum of four weeks before surgery. Written informed consent was obtained from parents or legal guardians, and age-appropriate assent was obtained from children wherever applicable.

Children undergoing emergency surgery, receiving polytherapy or having had recent changes in AED regimen within four weeks, experiencing acute febrile or metabolic seizures, classified as American Society of Anesthesiologists (ASA) physical status IV or V, or having incomplete perioperative records were excluded from the study. ASA physical status classification was assigned according to the ASA guidelines [8] and analyzed as a categorical variable (I, II, or III). Only children with well-controlled epilepsy without systemic involvement were categorized as ASA I.

Exposure definition

Exposure status was determined based on the type of preoperative AED therapy. Participants receiving levetiracetam monotherapy comprised the newer AED group, whereas those receiving phenytoin monotherapy comprised the conventional AED group. All patients continued their prescribed AED therapy throughout the perioperative period without modification, and the final preoperative dose was administered according to the routine dosing schedule.

Perioperative management

All participants underwent elective non-neurosurgical procedures, including general surgical, urological, and minor procedures, under general anesthesia. Anesthetic management included induction with intravenous agents and maintenance with inhalational and/or intravenous anesthetic agents according to institutional protocols. Standard intraoperative monitoring, including heart rate, blood pressure, oxygen saturation, and end-tidal CO₂, was applied in all cases.

Surgical duration and estimated blood loss were recorded and were comparable between groups. Continuous electroencephalographic and neuromuscular monitoring were not routinely performed. Standardized rescue protocols were followed throughout the perioperative period. Intraoperative seizures were managed with intravenous benzodiazepines, followed by second-line antiepileptic drugs if required. Hypotension was managed with intravenous fluid boluses and vasopressor support in accordance with standard pediatric anesthetic practice.

Sample size calculation

The sample size was calculated for the comparison of two independent proportions. Based on previously published neurosurgical randomized trials and meta-analyses, postoperative seizure rates were estimated at approximately 15% in patients receiving phenytoin and 6% in those receiving levetiracetam [3,9,10]. With a two-sided alpha level of 0.05 and a statistical power of 80%, the minimum required sample size was 177 participants per group to detect an absolute difference of 9%. However, due to feasibility constraints, only 100 participants were enrolled in each group. The observed absolute risk difference in this study was 6%, and overall event rates were lower than anticipated in elective non-neurosurgical procedures. This smaller-than-expected effect size likely reduced the statistical power to detect a significant difference in seizure incidence.

Operational definitions

Postoperative seizures were defined as any clinically observed convulsive or electrographic seizure occurring within 24 hours after surgery, consistent with perioperative seizure surveillance protocols used in neurosurgical and anesthetic outcome studies that classify early postoperative seizures within the first 24 hours as clinically significant events [11]. Hypotension was defined as a systolic blood pressure below the fifth percentile for age or any episode requiring vasopressor support, in accordance with established pediatric blood pressure reference standards and emergency pediatric life support criteria for abnormal hypotension [12]. Delayed emergence was defined as the failure to regain consciousness within 30 minutes after discontinuation of anesthetic agents, based on commonly accepted anesthesiology definitions that consider awakening beyond 20-30 minutes as abnormal recovery from general anesthesia [13]. Respiratory depression was defined as apnea, hypoventilation, or oxygen saturation below 92% requiring airway or ventilatory intervention, consistent with perioperative safety definitions used in anesthesia monitoring guidelines and the postoperative respiratory complication literature [14].

Outcome measures

The primary outcome was the occurrence of postoperative seizures within 24 hours following surgery. Postoperative seizure was defined as any clinically observed convulsive event during the first 24 postoperative hours. Secondary outcomes included preoperative seizure-free status for at least three months before surgery, intraoperative seizures, hypotension defined as systolic blood pressure below the fifth percentile for age or requiring vasopressor support, airway complications including laryngospasm, bronchospasm, or unplanned airway intervention, delayed emergence defined as failure to regain consciousness within 30 minutes after discontinuation of anesthetic agents, respiratory depression defined as oxygen saturation below 92% requiring airway or ventilatory support, recovery time measured in minutes from cessation of anesthesia to an appropriate response to verbal commands, and length of hospital stay measured in days.

Data collection and bias control

Data were collected prospectively using a structured case record form. Preoperative data included demographic characteristics, epilepsy duration, seizure-free status, AED type, and ASA grade. Intraoperative variables included type and duration of surgery, anesthetic technique, hemodynamic parameters, and perioperative complications. Postoperative monitoring was performed for 24 hours to document seizure occurrence, delayed emergence, respiratory depression, recovery time, and length of hospital stay. Because this was an observational cohort study, blinding to AED exposure was not feasible. However, consecutive recruitment helped minimize selection bias. Standardized operational definitions were used to reduce measurement bias. Outcome assessors were not involved in treatment decisions and were blinded to the specific study hypothesis. Data were coded before statistical analysis to ensure analytic blinding. Multivariable regression analysis was performed to adjust for potential confounding variables, including age, preoperative seizure-free status, AED group, and ASA grade.

Statistical analysis

Data were analyzed using Jamovi version 2.6.44 (The Jamovi Project, Sydney, Australia). Continuous variables are presented as mean ± standard deviation (SD), and categorical variables are presented as frequencies and percentages. Normality of continuous variables, including recovery time and hospital stay, was assessed using the Shapiro-Wilk test and demonstrated approximate normal distribution (p > 0.05), whereas age was not normally distributed (p < 0.05). Homogeneity of variance was assessed using Levene’s test. Accordingly, independent t-tests were used for between-group comparisons of normally distributed continuous variables (e.g., recovery time and hospital stay), while the Mann-Whitney U test was applied for non-normally distributed variables (e.g., age). Categorical variables were compared using the chi-square test or Fisher’s exact test, as appropriate.

Effect estimates for categorical outcomes are reported as odds ratios (ORs) with 95% confidence intervals (CIs). For continuous outcomes, mean differences with 95% CIs and Cohen’s d effect sizes were calculated. Absolute risk reduction (ARR) was calculated as the difference in event proportions between groups, and the number needed to treat (NNT) was derived as the reciprocal of ARR. Multivariable logistic regression analysis was performed to identify independent predictors of postoperative seizures. Adjusted ORs with 95% CIs were reported. Multicollinearity was assessed using the variance inflation factor (VIF), with values < 2 considered acceptable. Model calibration was evaluated using the Hosmer-Lemeshow goodness-of-fit test. A p-value < 0.05 was considered statistically significant.

Ethical considerations

Institutional Ethics Committee approval (127/CM/GMC/IECBMHR/2025) was obtained before study initiation. Written informed consent and age-appropriate assent were obtained from participants and their guardians. As this was an observational study, no changes were made to prescribed AED therapy. Participant confidentiality was maintained throughout the study.

## Results

A total of 200 children were analyzed, with 100 participants each in the levetiracetam and phenytoin groups. Baseline demographic and perioperative characteristics were largely comparable between groups. The mean age was 8.91 ± 5.37 years in the levetiracetam group and 9.74 ± 4.99 years in the phenytoin group (p = 0.31). The proportion of male participants was similar (51% vs. 56%, p = 0.47). ASA physical status distribution was comparable between groups, with no statistically significant differences observed across ASA categories (all p > 0.05). However, a significantly greater proportion of patients receiving levetiracetam were seizure-free for ≥ 3 months preoperatively than those receiving phenytoin (79% vs. 54%, p < 0.001), indicating a baseline imbalance in seizure control (Table 1).

**Table 1:** Baseline characteristics and seizure control outcomes among patients receiving levetiracetam and phenytoin ^***^P < 0.001 Continuous variables are presented as mean ± SD. Age, which was not normally distributed, was compared using the Mann-Whitney U test. Categorical variables are presented as frequency (n) and percentage (%) and were compared using the chi-square test. A p-value < 0.05 was considered statistically significant SD: standard deviation; ASA: American Society of Anesthesiologists

Variable	Levetiracetam (n = 100)	Phenytoin (n = 100)	Statistic	P-value
Age, years, mean ± SD	8.91 ± 5.37	9.74 ± 4.99	4525	0.31
Male sex, n (%)	51 (51%)	56 (56%)	0.502	0.47
ASA I, n (%)	25 (25.0%)	30 (30.0%)	0.454	0.5
ASA II, n (%)	39 (39.0%)	37 (37.0%)	0.05	0.818
ASA III, n (%)	36 (36.0%)	33 (33.0%)	0.13	0.717
Seizure-free for ≥ 3 months, n (%)	79 (79%)	54 (54%)	14	<0.001^***^

Postoperative seizures occurred in 6% of the levetiracetam group and 12% of the phenytoin group (OR: 0.47; 95% CI: 0.17-1.29; p = 0.138). Although the proportion was lower in the levetiracetam group, the difference did not reach statistical significance. Hypotension occurred in 10% of patients in the levetiracetam group and 18% in the phenytoin group (OR: 0.51; 95% CI: 0.23-1.15; p = 0.103). Airway complications were observed in 4% and 7% of patients, respectively (OR: 0.55; 95% CI: 0.16-1.97; p = 0.352). Intraoperative seizures occurred in 1% of the levetiracetam group and 4% of the phenytoin group (OR: 0.24; 95% CI: 0.03-2.20; p = 0.174), while respiratory depression occurred in 3% and 5% of patients, respectively (OR: 0.59; 95% CI: 0.14-2.58; p = 0.47). Delayed emergence was significantly lower in the levetiracetam group compared with the phenytoin group (7% vs. 19%; OR: 0.32; 95% CI: 0.13-0.78; p = 0.012), representing a statistically significant and clinically relevant difference (Table 2).

**Table 2:** Comparison of postoperative and perioperative adverse outcomes between levetiracetam and phenytoin groups ^*^P < 0.05 Group comparisons were performed using the chi-square test. Effect size is reported as OR with 95% CI, comparing levetiracetam with phenytoin. ARR and NNT are also presented. A p-value < 0.05 was considered statistically significant OR: odds ratio; CI: confidence interval; ARR: absolute risk reduction; NNT: number needed to treat

Outcome	Levetiracetam (n = 100), n (%)	Phenytoin (n = 100), n (%)	OR (95% CI)	P-value	ARR	NNT
Postoperative seizure	6 (6%)	12 (12%)	0.47 (0.17–1.29)	0.138	6%	17
Hypotension	10 (10%)	18 (18%)	0.51 (0.23–1.15)	0.103	8%	13
Airway complication	4 (4%)	7 (7%)	0.55 (0.16–1.97)	0.352	3%	34
Intraoperative seizure	1 (1%)	4 (4%)	0.24 (0.03–2.20)	0.174	3%	34
Delayed emergence	7 (7%)	19 (19%)	0.32 (0.13–0.78)	0.012^*^	12%	9
Respiratory depression	3 (3%)	5 (5%)	0.59 (0.14–2.58)	0.47	2%	50

Mean surgical duration was comparable between groups (105 ± 41.9 minutes in the levetiracetam group vs. 107 ± 45.2 minutes in the phenytoin group; p = 0.74). Recovery time was significantly shorter in the levetiracetam group (34.5 ± 9.1 minutes) compared to the phenytoin group (51.4 ± 12.8 minutes), with a mean difference of −16.9 minutes (95% CI: −20.0 to −13.8; p < 0.001). Similarly, the length of hospital stay was significantly shorter in the levetiracetam group (2.95 ± 0.86 days vs. 4.83 ± 1.39 days), with a mean difference of −1.88 days (95% CI: −2.18 to −1.58; p < 0.001) (Table 3).

**Table 3:** Comparison of perioperative time parameters and hospital stay between levetiracetam and phenytoin groups ^***^P < 0.001 Continuous variables are presented as mean ± SD. Between-group comparisons were performed using the independent t-test after confirming normality. Mean difference represents the value in the levetiracetam group minus the value in the phenytoin group. Effect size is expressed as Cohen’s d. A p-value < 0.05 was considered statistically significant SD: standard deviation; CI: confidence interval

Variable	Levetiracetam, mean ± SD	Phenytoin, mean ± SD	Mean difference	95% CI	Cohen’s d	P-value
Surgery duration, minutes	105 ± 41.9	107 ± 45.2	−2.0	−14.0 to 10.0	0.05	0.74
Recovery time, minutes	34.5 ± 9.1	51.4 ± 12.8	−16.9	−20.0 to −13.8	1.52	<0.001^***^
Hospital stay, minutes	2.95 ± 0.86	4.83 ± 1.39	−1.88	−2.18 to −1.58	1.60	<0.001^***^

Multivariable logistic regression analysis was performed to evaluate independent predictors of postoperative seizures, adjusting for age, preoperative seizure-free status (≥ 3 months), AED group, and ASA grade (Table 4). Age was not significantly associated with postoperative seizures (adjusted OR: 0.99; 95% CI: 0.90-1.09; p = 0.847). Preoperative seizure-free status showed a protective trend (adjusted OR: 0.42; 95% CI: 0.15-1.16; p = 0.094), but it did not reach statistical significance. AED group was not independently associated with postoperative seizures (adjusted OR: 1.72; 95% CI: 0.59-5.01; p = 0.32). ASA grade was also not significantly associated with the outcome (adjusted OR: 0.98; 95% CI: 0.52-1.82; p = 0.936). Variance inflation factor (VIF) values were < 2 for all variables, indicating no significant multicollinearity. None of the variables demonstrated a statistically significant independent association with postoperative seizures after adjustment (Table 4).

**Table 4:** Multivariable logistic regression analysis of the predictors of postoperative seizures Multivariable logistic regression analysis was performed to identify independent predictors of postoperative seizures. Results are presented as regression coefficient (β), SE, and adjusted ORs with 95% CIs. Variables included in the model were age, preoperative seizure-free status, AED group, and ASA grade. A p-value < 0.05 was considered statistically significant. SE: standard error; OR: odds ratio; CI: confidence intervals, ASA: American Society of Anesthesiologists; AED: antiepileptic drug

Predictor	β	SE	Adjusted OR (95% CI)	P-value
Age	−0.010	0.05	0.99 (0.90–1.09)	0.847
Seizure-free for ≥ 3 months	−0.869	0.519	0.42 (0.15–1.16)	0.094
Phenytoin vs. levetiracetam	0.542	0.545	1.72 (0.59–5.01)	0.32
ASA grade, ordinal	−0.026	0.32	0.98 (0.52–1.82)	0.936

## Discussion

This study provides a focused evaluation of the influence of preoperative antiepileptic drug (AED) therapy on perioperative seizure control and anesthetic recovery in children undergoing elective surgery. Three clinically relevant observations were identified. First, children receiving levetiracetam had a higher proportion of preoperative seizure-free status compared with those receiving phenytoin. Second, perioperative anesthetic morbidity, particularly delayed emergence, was significantly lower in the levetiracetam group. Third, postoperative recovery metrics, including time to awakening and duration of hospital stay, were significantly improved with levetiracetam. In contrast, the difference in postoperative seizure incidence did not reach statistical significance after adjustment for confounding variables. These findings suggest that differences between the two drugs may be more evident in perioperative recovery characteristics rather than seizure prevention.

Baseline demographic and surgical comparability between groups supports the internal validity of these observations and reduces the likelihood that differences are attributable to case mix or operative complexity. However, a significant baseline imbalance was observed in preoperative seizure-free status, with a higher proportion of seizure-free children in the levetiracetam group. As preoperative seizure control is a well-recognized determinant of perioperative seizure risk, this represents an important potential confounding factor. Children who are seizure-free before surgery inherently have a lower likelihood of postoperative seizures, irrespective of the AED used. Although this variable was included in the multivariable regression model, residual confounding cannot be excluded due to the observational design and the limited number of outcome events. Therefore, the findings should be interpreted as associations rather than causal relationships.

Phenytoin and levetiracetam differ substantially in their pharmacological profiles, which may contribute to differences in perioperative outcomes. Phenytoin is associated with hepatic enzyme induction and significant drug-drug interactions that can alter anesthetic metabolism [4]. Intravenous phenytoin loading has been associated with hypotension and arrhythmias, whereas chronic oral therapy typically has minimal intraoperative cardiovascular effects. In contrast, levetiracetam has minimal protein binding, negligible hepatic metabolism, and limited drug interactions, resulting in a more predictable pharmacokinetic profile [5]. In addition to pharmacokinetic differences, AEDs may influence perioperative airway and respiratory outcomes. Enzyme induction by phenytoin may alter anesthetic drug metabolism, potentially contributing to variability in anesthetic depth and delayed recovery. Interactions between AEDs and anesthetic agents may enhance sedation and respiratory depressant effects, which can predispose to airway complications. In contrast, levetiracetam’s minimal interaction profile may result in more stable perioperative respiratory and anesthetic responses.

Previous pediatric studies in acute and neurocritical settings have demonstrated that levetiracetam provides seizure control comparable to or better than phenytoin, with fewer adverse effects [2,4,5]. In neurosurgical populations, levetiracetam has been associated with lower perioperative seizure rates and improved tolerability [3,8]. However, these studies primarily focused on emergency or high-risk neurosurgical contexts. The present study extends these observations to elective non-neurosurgical procedures, where perioperative physiology and seizure risk differ.

Although a numerically lower rate of postoperative seizures was observed in the levetiracetam group (6% vs 12%), this difference did not achieve statistical significance after multivariable adjustment. This finding is consistent with contemporary evidence suggesting broadly comparable antiseizure efficacy between levetiracetam and phenytoin or fosphenytoin in pediatric populations [15]. Similarly, secondary analyses in pediatric traumatic brain injury cohorts have shown no clear superiority of one agent over the other for seizure prevention [16]. These findings indicate that baseline disease characteristics, particularly seizure control status, may have a greater influence on perioperative seizure risk than the choice of AED.

The most clinically meaningful finding in this study was the significant reduction in delayed emergence and faster recovery associated with levetiracetam. Delayed awakening prolongs post-anesthesia care requirements and may reflect altered anesthetic metabolism or residual sedative effects. Phenytoin’s enzyme-inducing properties may contribute to altered anesthetic clearance, whereas levetiracetam’s minimal interaction profile may facilitate more predictable recovery. Supporting this, pooled analyses have reported lower rates of cardiorespiratory adverse events with levetiracetam compared with phenytoin [15], and clinical reviews have highlighted improved tolerability with levetiracetam in acute seizure management [17]. The shorter hospital stay observed in this study likely reflects these recovery advantages and has implications for healthcare resource utilization.

Multivariable regression analysis identified preoperative seizure-free status as an important factor associated with postoperative seizure risk, demonstrating a protective trend, although statistical significance was not reached. In contrast, the AED type was not independently associated with postoperative seizures. This finding is consistent with prior evidence indicating that intrinsic patient factors and baseline seizure control play a more substantial role in determining perioperative seizure risk than pharmacological differences alone [11,15,16]. Subgroup analysis based on preoperative seizure-free status was not performed due to limited event numbers and reduced statistical power.

The present study contributes to the existing literature by specifically examining elective non-neurosurgical pediatric procedures, a relatively underrepresented area. By demonstrating comparable seizure prevention but improved recovery characteristics with levetiracetam, the findings suggest that AED selection in the perioperative setting may be guided not only by seizure control but also by safety and recovery profiles.

Strengths

This study has several methodological strengths. It employed a prospective comparative cohort design with clearly defined exposure groups and standardized outcome measures. Comprehensive perioperative data, including seizure-related and anesthetic outcomes, were systematically collected. The use of multivariable logistic regression allowed for adjustment for key confounding variables, particularly preoperative seizure-free status, age, AED group, and ASA grade. Additionally, the study specifically focused on elective non-neurosurgical pediatric procedures, an area that remains underrepresented in the existing literature. Complete follow-up for all participants further enhances the reliability and internal validity of the findings.

Limitations

This study has several limitations, which should be considered while interpreting the results. First, the non-randomized observational design introduces the possibility of selection bias and residual confounding. A significant baseline imbalance in preoperative seizure-free status between groups represents a major limitation, as this variable is a strong predictor of postoperative seizure risk. This baseline imbalance represents the most important methodological limitation of the study. Although adjustment was performed using multivariable analysis, residual confounding cannot be completely excluded. Second, the sample size was smaller than initially calculated, which may have reduced the statistical power to detect modest differences in postoperative seizure incidence, increasing the likelihood of a type II error. Third, intraoperative seizure detection was based on clinical observation without continuous electroencephalographic monitoring, which may have resulted in an underestimation of subclinical events.

Fourth, anesthetic techniques and perioperative management were not fully standardized, and variability in anesthetic practice may have influenced recovery outcomes. Fifth, serum antiepileptic drug levels were not measured, limiting the assessment of pharmacokinetic variability and its potential impact on perioperative outcomes. Finally, postoperative outcomes were assessed only within the first 24 hours, precluding evaluation of longer-term seizure control, neurocognitive outcomes, and functional recovery. These limitations should be considered when interpreting the findings, and further studies with larger sample sizes, randomized designs, and advanced monitoring are warranted.

Clinical implications

Perioperative management of pediatric epilepsy necessitates careful evaluation of seizure control, pharmacokinetic stability, drug-drug interactions, and recovery profiles. In the studied cohort, levetiracetam and phenytoin resulted in similar postoperative seizure incidence after adjustment for confounding variables. Notably, levetiracetam was associated with a significant reduction in delayed emergence, as well as faster recovery and a shorter hospital stay. These results indicate that when seizure control is equivalent, choosing an antiepileptic drug with fewer pharmacologic interactions and more predictable recovery characteristics may offer perioperative benefits. A reduction in delayed emergence could decrease post-anesthesia care unit occupancy and enhance perioperative workflow efficiency. Although drug selection should be individualized, levetiracetam appears to be a suitable perioperative option in elective pediatric surgical contexts, especially when rapid recovery and hemodynamic stability are prioritized.

Future research directions

Future research should prioritize large, multicenter randomized controlled trials that compare newer and conventional AEDs in elective pediatric surgical populations. Such studies are essential for minimizing confounding variables and establishing causal relationships. Sufficient sample sizes will be required to detect modest differences in postoperative seizure risk and to identify infrequent perioperative complications.

The incorporation of continuous electroencephalographic monitoring would improve the detection of subclinical seizures and enhance the accuracy of outcome assessment. Extended follow-up periods are necessary to evaluate long-term seizure recurrence, neurocognitive outcomes, and functional recovery. Furthermore, comparative cost-effectiveness analyses and evaluation of other newer AEDs would offer broader guidance for perioperative drug selection. These investigations would facilitate the development of standardized, evidence-based protocols for the perioperative management of pediatric epilepsy.

## Conclusions

Levetiracetam and phenytoin demonstrated comparable postoperative seizure incidence after adjustment for confounding variables, particularly preoperative seizure-free status, in children undergoing elective surgery. However, levetiracetam was associated with improved perioperative recovery, including reduced delayed emergence, shorter recovery time, and decreased length of hospital stay. These findings suggest that while antiseizure efficacy may be similar, differences in pharmacological profiles may influence perioperative safety and recovery outcomes. Given the observational design and baseline imbalance, the results should be interpreted as associations rather than causal relationships, and further randomized studies are warranted.
